# Perspectives of autistic adults on the strategies that help or hinder successful conversations

**DOI:** 10.1177/23969415221101113

**Published:** 2022-05-20

**Authors:** Kate Silver, Sarah Parsons

**Affiliations:** The Autism Community Research Network @Southampton [ACoRNS] and the Centre for Research in Inclusion, Southampton Education School, 7423 University of Southampton, UK

**Keywords:** Autism, autistic perspectives, conversation, trait knowledge, interactional expertise

## Abstract

**Background & aims:**

There is increasing recognition of the importance of challenging deficit-focused, medical model approaches to supporting autistic people in daily life, however there is a lack of inclusion of autistic perspectives to inform approaches that may empower autistic people in conversations.

**Methods:**

This multiple case study used a participatory approach to explore the conversation experiences and exchange in dyads of five autistic and five non-autistic adults over four to 12 months. The study was grounded in the perspectives of autistic people through a series of semi-structured interviews, observations, reflective conversations, and diary records.

**Results:**

The findings focus on autistic participants’ existing knowledge of conversations that they reported could be useful to them, including the communication environment, and type and structure of talk. The study also helped participants to identify and use previously unrecognised metacognitive abilities (what they already knew about conversations) within naturalistic interactive contexts.

**Conclusions:**

These findings provide novel insights as to how the ‘interactional expertise’ of non-autistic people could be strengthened to enable the effective contribution of the voices of autistic people in everyday conversations.

**Implications:**

The identification and use of successful conversation strategies identified by autistic adults gave them a greater sense of empowerment within the conversation based on their accounts of their experiences. Understanding these strategies has valuable implications for staff training, for working with families and for learning by autistic adults.

## Introduction

### A note about terminology

There have been changes over time in the way that people with a diagnosis of autism choose to refer to themselves and their diagnosis (Kenny et al., 2016). When working with individual people we always use the term preferred by the person themselves. Within this paper we use ‘autistic’ or ‘on the autism spectrum’ to refer to people with a diagnosis of autism and ‘non-autistic’ to refer to people without a diagnosis of autism. This approach also aligns with recommendations from the autistic community about the use of non-ableist language (Bottema-Beutel et al., 2021).

### Rationale for the study

Difficulties in the social world are well described in autobiographical accounts by autistic people (e.g., Grandin, 1995; Jackson, 2003; Williams, 1992). Specifically, experiences of anxiety in social situations are described in studies with young autistic people (e.g., Carrington & Graham, 2001; Humphrey & Lewis, 2008; Jones et al., 2001; Knott et al., 2006) and autistic adults (Beardon & Edmonds, 2007; Wittemeyer et al., 2015). Social anxiety is reported as being at a higher level in autistic people than in comparison groups when a range of assessment measures are used (Bejerot et al., 2014; Bellini, 2004; Galanopoulos et al., 2014; Simonoff et al., 2008; Spain et al., 2016; White et al., 2006). Although the cause of the anxiety is highly individualised, it has been argued by some that the pressure to change and to conform to the expectations of a majority non-autistic world may cause young autistic people to experience increased anxiety (Crane et al., 2019; Trembath et al., 2012) and may be detrimental to their sense of well-being (Cribb et al., 2019). Indeed, ‘camouflaging’ (hiding behaviours associated with their autism in social situations) and finding ways to prevent others from seeing their social difficulties is described by autistic adults as being mentally, physically, and emotionally draining (Hull et al., 2017).

The traditional way of responding to such difficulties, and supporting autistic people to navigate the social world, has been to place an emphasis on the autistic person's skills, understanding and capabilities. Such approaches often comprise different methods or approaches to ‘social skills training’ and focus on changing the communication, understanding and behaviour of the autistic person (e.g., Bishop-Fitzpatrick et al., 2014; Rao et al., 2008; Reichow & Volkmar, 2010; Williams-White et al., 2007). Reviews report variable outcomes and methodological concerns of social skills training in its various forms, including a lack of well-controlled designs and follow-up data (Bottema-Beutel et al., 2018; Gates et al., 2017; Reichow et al., 2013). However, and perhaps more crucial than these methodological concerns, is a more fundamental issue with the premise for the focus of any intervention i.e., the difficulties of the autistic person themselves. Much of the social skills literature tends to report a ‘one size fits all’ approach within a deficit model of teaching in contrived contexts, often informed or led by the perspective of a non-autistic person (usually a researcher or clinician) and, increasingly outdated, cognitive theories of autism such as ‘theory of mind’ (Bottema-Beutel et al., 2018). Such approaches tend to assume that the problem with communication lies within the autistic person, rather than in an interaction that includes other people.

The use of these kinds of approaches is described as a ‘normalisation agenda’ by Milton and Moon (2012, p. 1). Indeed, Milton (2014) argues that autistic people are taught social rules as if the rules are more fixed and static than they actually are in daily life, so that social skills learned as isolated rules may be of little help to autistic people in real-world contexts and may actually contribute to social anxiety reported above due to increased cognitive demands and pressure to behave inauthentically (Bottema-Beutel et al., 2018). Furthermore, Milton (2012, p. 883) reminds us that communication is a dialogue between people and that difficulties in understanding arise not because the autistic person is inherently deficient but because of a ‘double empathy problem’. In other words, that autistic people and non-autistic people can find it difficult to understand the perspectives of each other, and it is this difficulty, located in the interaction between people where one is a member of a dominant social group and the other a member of a marginalised social group, that can lead to misunderstandings.

Consequently, there is a call to move towards the use of naturalistic (not contrived) settings to enable autistic people to learn about social information (Chevallier et al., 2013), in ways that respect their authenticity and preferences (Bottema-Beutel et al., 2018), and for the focus to be on how social knowledge can be used across contexts and with greater support from peers (Bishop-Fitzpatrick et al., 2014; Flynn & Healy, 2012). In addition, the use of the perspectives, strengths, knowledge and needs of autistic people as starting points for support strategies is increasingly recognised as important but also a gap in the field (Pellicano et al., 2014). As Milton (2014, p. 800) observes:

‘In order to build bridges and practice languages, at the very least, autistic people need to be listened to.’

When autistic perspectives are taken as a starting point, this can reveal valuable information about existing communication strengths (or ‘practice language’ in Milton’s [2014, p. 800] terms) that may also counter longstanding assumptions about helpful strategies. For example, a small-scale study by Silver and Parsons (2015) explored the strategies used by three autistic adults in managing social conversations in ways that were helpful to them. Noticing the unusual, consideration of the potential impact of others’ behaviour on them personally and guessing the intention of others were identified as useful strategies. Interestingly, basing social judgments on the emotions identified in facial expressions of others was not identified as a useful strategy by the participants, even though this is a widely used approach in many social skills interventions (e.g., Baghdadli et al., 2013).

Nevertheless, Silver and Parsons (2015) was a small-scale study and overall, there is very little published evidence, to the best of our knowledge, about *how* the strengths and existing abilities of autistic people can be meaningfully recognised and understood to become the starting point for personalised strategies to support social interactions. Moreover, there is very little focus placed on the role of the person with whom an autistic person is communicating as either a help or hindrance to successful communication. Therefore, this study aimed to develop a more naturalistic, strengths-based, and inclusive approach to supporting autistic people to manage social interactions in ways that empower them. Specifically, the objectives were to enable autistic people to identify communication strategies that work for them based on their existing knowledge; and to work with communication partners (who were all non-autistic) to help them discover how their own communication may help or hinder the participation and engagement of the autistic person. Due to the richness of the data collected we focus here on the findings from the first study objective on the existing knowledge of autistic people and will report the findings from the non-autistic communication partners separately in due course. Specifically, the research question addressed by this study was: What is useful for autistic adults to know about conversations?

## Methods

### Research design

A multiple case study informed by participatory approaches was used to learn directly from autistic adults about what they already know, and the strategies they may usefully employ in conversations to enable the conversation to go well from their perspective. Because communication involves more than one person and there is a need for learning to consider the interactive context (Bottema-Beutel et al., 2018), the focus of each case study was on the conversation exchanges between adult communication partners who knew each other well. The focus was on understanding the autistic perspective of what both participants within the dyad (autistic and non-autistic) could think, say, or do to maximise the contribution of thoughts and knowledge of the autistic participant.

There were two main phases to the study. Phase 1 was designed to explore the autistic participants’ perspectives of what may have contributed to a positive or negative conversation experience. This included finding out what they felt had influenced whether their own contribution to the conversation was successful (on their own terms). Phase 2 built upon what was learned in Phase 1 such that when an ability or particular knowledge was found to be useful to one participant, its value was subsequently explored with others. Similarly, where autistic participants had accessed their own existing useful knowledge about successful conversations in Phase 1, this knowledge was discussed further, and applied, in Phase 2.

### Participants and recruitment

During the data collection period, the first author was employed as a senior manager by a charitable organisation that provides social care and educational provision for autistic children, young people, and adults in England. Study participants were recruited via staff at this organisation who explained the purpose and potential benefits of the study, and what their involvement would look like in practice. Potential participants then had opportunity to find out more about the study from the researcher (first author), before reviewing and signing a consent form. All autistic participants had the ability and desire to be involved in significant conversations, particularly to plan, problem-solve, and to effectively convey information important to them. After agreeing to take part, each autistic participant invited a non-autistic person, known to them, to join them as their communication partner in the study. These participant dyads were all people who wished to gain from participation in the study, having experienced difficult communication encounters in the past.

Five autistic participants agreed to take part in phase 1, with each asking a communication partner to join them in the study. Three participants chose to continue their participation into a second phase of the study. A summary of the participants, including formal diagnosis of the autistic participant based on their records, and length of participation in the study, is shown in Table 1. All names are pseudonyms.

**Table 1.** Background details of participants.

	Autistic person				Communication partner		
Dyad	Pseudonym	Sex	Age	Diagnosis from formal records	Sex	Role / relationship	Length of study participation
1	Chloe Lived in Supported residential setting since 1993 and accesses the community independently	Female	42	Asperger Syndrome (1988)	Female	Support worker Supporting Chloe for 10 years	5 months Phase 1 of the study only
2	Carl Lived in Supported Living since 2009 and accesses the community independently	Male	34	Autism Spectrum Disorder (1993)	Female	Support worker Known to Carl for 1 year, providing some direct support	4 months Phase 1 of the study only
3	Ruth Lives in the family home, attends mainstream college and does voluntary work with children	Female	21	Autism Spectrum Disorder (1995)	Female	Teacher Known to Ruth for 5 years, overseeing her voluntary placements	12 months Phases 1 and 2 of the study
4	Lee Lives in the family home and attends an autism specialist college	Male	18	Autism Spectrum Disorder (2012)	Female	Speech and language therapist Working regularly with Lee	12 months Phases 1 and 2 of the study
5	Cait Lives alone and receives some outreach support, works in a supermarket	Female	40	Asperger Syndrome (date unknown)	Female Female	Support worker (Outreach) Supporting Cait for 3 years Cait's mother who sees Cait several times a week	6 months Phases 1 and 2 of the study Phase 1 and 2 of the study

### Procedure

**Figure 1.**
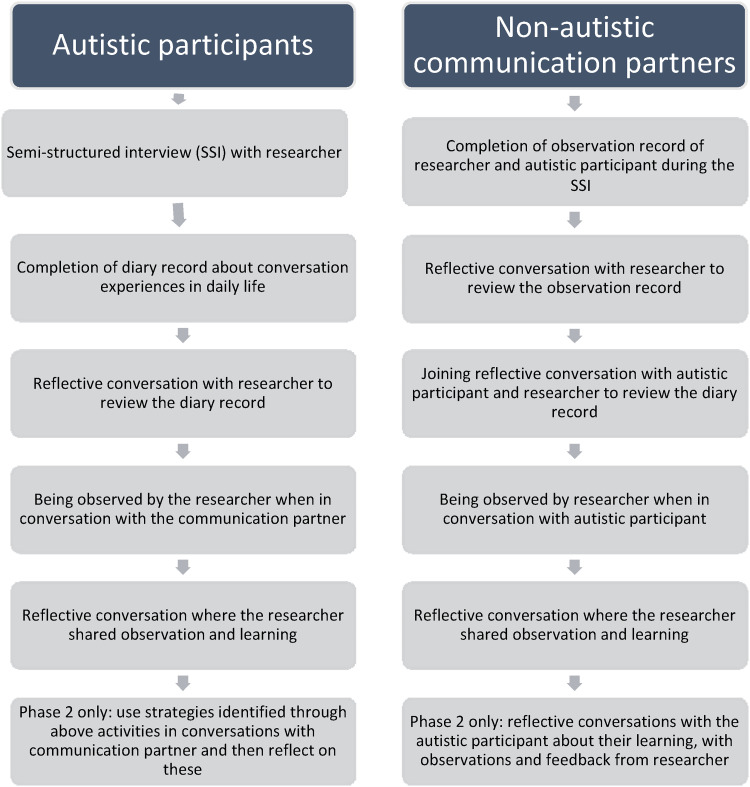
Summary and sequence of the methods for the autistic participants and their non-autistic communication partners.

The research followed a series of steps summarised in Figure 1 that can be characterised as an action-oriented sequence of reflections on conversations that had taken place, active involvement in naturally occurring conversations, and observations of conversations. Autistic participants were involved in semi-structured interviews, reflective conversations, observations of conversations with their communication partner, and encouraged to keep diary records of everyday conversations that they had been involved in with their communication partner and with others not directly involved in the study. This meant there was a range of conversations noted down and reflected upon. The focus of the interviews was to explore the perspectives of the autistic people of conversations that had taken place and what may have contributed to a positive or negative conversation experience from their perspective. This included their preferences and knowledge about what was useful to them in supporting their contribution to conversations and what they felt happened (e.g., what the communication partner said and did and the impact on them) when conversations did not go well. These interviews were based on a topic guide, but questions or requests for information were not always phrased in the same way or in the same order for all participants. This was so that the phrasing of questions and probes could be adapted to match the understanding of each autistic participant and ensure that the interview was conducted in the same form as a natural conversation, where one topic naturally leads to another.

The personalized diary records were used by autistic participants to record conversation experiences when they had taken place and were intended as a scaffold for discussions. The semi-structured interview to explore the diary record was structured around what was written, so this interview may be more accurately described as a reflective conversation; the focus followed whatever the participants found important and wanted to talk about. The reflective conversations enabled the autistic participant, to reflect on what worked well for them in conversation, from their own perspective (what they knew and what they could do to help themselves) and also what approaches or actions from the conversation partner were un/helpful to them.

The role of the communication partners was to initially observe the autistic person in conversation with the researcher, and then to reflect on what was working well to enable contribution of the autistic participant and two-way dialogue between the conversation partners, by making some observation notes and discussing these with the researcher. The communication partner then joined in the reflective discussion of the participant's diary record and was also observed by the researcher in conversation with the autistic person. The researcher then facilitated a reflective conversation with the autistic person and their communication partner about her observations of their conversation and their learning and feedback for each other. This sequence of interviews and reflective discussions was designed to identify with the autistic participants the successful strategies they were already using (often unconsciously) in conversation (with both the communication partner and others) and to make these strategies more explicit for them, and to identify things that the communication partners said and did that were helpful and unhelpful to the conversation.

The autistic participants chose where and when the interviews were to take place so that they felt comfortable, and the location also needed to be convenient for the communication partner. For example, Lee worked in the kitchens at school and did not want the interview to take place immediately after his work, nor in the school environment, and so meetings were always scheduled in the morning, in a nearby college. It was important that the room felt right to the autistic participants; for example, having chosen a room at the college it had a clock that could be heard ticking, and this was distracting for Lee and therefore, removed. The interviews were recorded using a digital voice recorder. Each autistic participant was invited to take the recorder, so they could switch the recorder on and off or tell the researcher when to do this, to have control of the interview and the recording.

The purpose of each meeting or activity was explained and checked with all participants to reduce the possibility of misunderstanding and any associated anxiety, and to check ongoing consent. At the end of each phase of the study, each participant had the opportunity to reflect on what had been learned and contribute their ideas about how that learning could be applied in the future. During phase 2, participants used their learning from phase 1 in conversation with their communication partners, and in conversations important to them with other people. Two autistic participants subsequently chose to be involved in staff training based on their participation and these conversations.

### Ethics

Considerable care was taken throughout the study to ensure all participants understood the focus of the project and their roles within it. All formally required documents (information sheet, consent form) were written in Plain English and supported, where needed, with meetings and conversations to answer any questions. All participants provided fully informed consent and were asked to sign a consent form prior to the study commencing. The project was reviewed and approved by the Faculty of Social Sciences ethics committee at the University of Southampton (Reference: 17940).

### Analysis

Transcripts of the semi-structured interviews, reflective conversations, observations, and diary records were reflexively thematically analysed using the step-by-step methods described by Braun and Clarke (2006, 2020) and Miles et al. (2014). This thematic approach uses an in depth understanding of the data and selectively collates data to create inductive inference, searches for patterns and clusters of meaning, and then draws inferences based on the links between the data segments. The analysis focused on what was helpful and unhelpful to autistic participants in conversations. Consequently, some codes were theory driven, ‘top down’ and deductive, specifically relating to what the research question asked and to exploration of known theory and practice relating to autistic people and to conversation, and provided a ‘start list’ (Miles et al., 2014, p. 81). This start list was informed by understanding the cognitive differences relating to autism, for example the code ‘Existing knowledge of own response to an interaction’ was used in response to the research question and differences typically associated with autism, to document apparent knowledge based on discussions of self-awareness and metacognition (e.g., Frith, 2012; Grainger et al., 2014) (see examples in Table 2). The start list was also influenced by the researcher's personal theory (based on learning directly from autistic people during clinical experience) that autistic people have knowledge useful to conversation, but that this is not always readily accessed by them or used in conversations. Other codes were identified during the process of analysis as is common and expected with a reflexive thematic approach (Braun & Clarke, 2020). These codes had not been anticipated when devising the ‘start list’ of deductive codes, and so were inductive, ‘bottom up’ codes driven by the data. Use of the inductive codes alongside the deductive codes avoided any attempt to force fit the data into pre-existing ideas (Miles et al., 2014), meaning that new factors and patterns identified through the analysis process could be responded to. For example, knowing the ‘type of conversation’ had not been anticipated as significant when starting analysis with the deductive codes, but when the analysis showed that this was important to more than one participant it became an early inductive code identified within the data. It is very common for qualitative coding to draw upon both deductive and inductive ‘forces’ in this way (Saldaña, 2013, p. 54). Codes that could be linked together were then clustered under an ‘overarching theme’ (Braun & Clarke 2006, p. 89). On completion of the analysis all the data was revisited and emerging interpretations arising from coding were checked with the participants to ensure that these made sense to the participants and that the codes were relevant to real conversation in the real world. A full account of the analysis, including extensive examples, can be found in the thesis upon which this paper is based (Silver, 2019).

**Table 2.** Deductive ‘start list’ codes relating to the research question: *what is useful for autistic adults to know about conversations?*.

‘Start list’ Code	Definition	Excerpt (taken from autistic participant)
Existing knowledge of unsuccessful interaction (EKU) Autism context – difference in social understanding and difficulty in social interaction evidenced	Comment on involvement in bad/unsuccessful conversations or interactions	Carl: When someone is talking negatively, I would see that as a bad conversation Lee: too many interruptions, it's not going well
Existing knowledge of good/successful interaction (EKG) Autism context – difference in social understanding and difficulty in social interaction evidenced	Comment on experiences of good and successful conversation. Comment on experiences of good and successful conversations. Code as EKG when the conversation described was clearly good from the perspective of the autistic participant	Cait: she was helpful. We got it solved Ruth: I understood what she was saying, it was very clear
Existing knowledge of response to an interaction (EKR) Code quickly became renamed as: Existing knowledge of their response to an unsuccessful interaction, as response was only described in relation to situations described as ‘bad’ (or a word carrying a similar meaning) and coded as unsuccessful (EKUR)	Comment where the autistic person talks about something that they thought, did or felt in response to something said or done to them in the conversation or interaction	Lee: I wanted to hit him in the face Ruth: there's a little voice in my head that said you don't have to be in this
A physical response EKPh I looked for this based on my practice knowledge, so have categorised it ‘deductive’	Comment on something happening within the body when the conversation or interaction is not ‘right’. The definition began as a ‘physical response’ but was expanded to include ‘sensation’ when sensation became a pattern in the data set	Chloe: my jaw started to stress out Ruth: the sensation I got in my body was quite tensed, I was kind of stuck against the wall
Vulnerability Categorised as ‘deductive’ because I looked for it in relation to other research questions in the research Autism context – vulnerability is well reported	Comment on vulnerability in interaction, whether vulnerability is experienced, and what vulnerability means to the person	Lee: they tried to twist the questions Ruth: Vulnerable, erm erm, I know how to describe it… erm it's really hard… possibly when you’re in classes you’re vulnerable there cos you don't know what to say and you’re struggling to find the words like I am at the moment

NB The notes regarding the autism context are reminders that the code is important in relation to theory and knowledge about autism.

## Results

**Figure 2.**
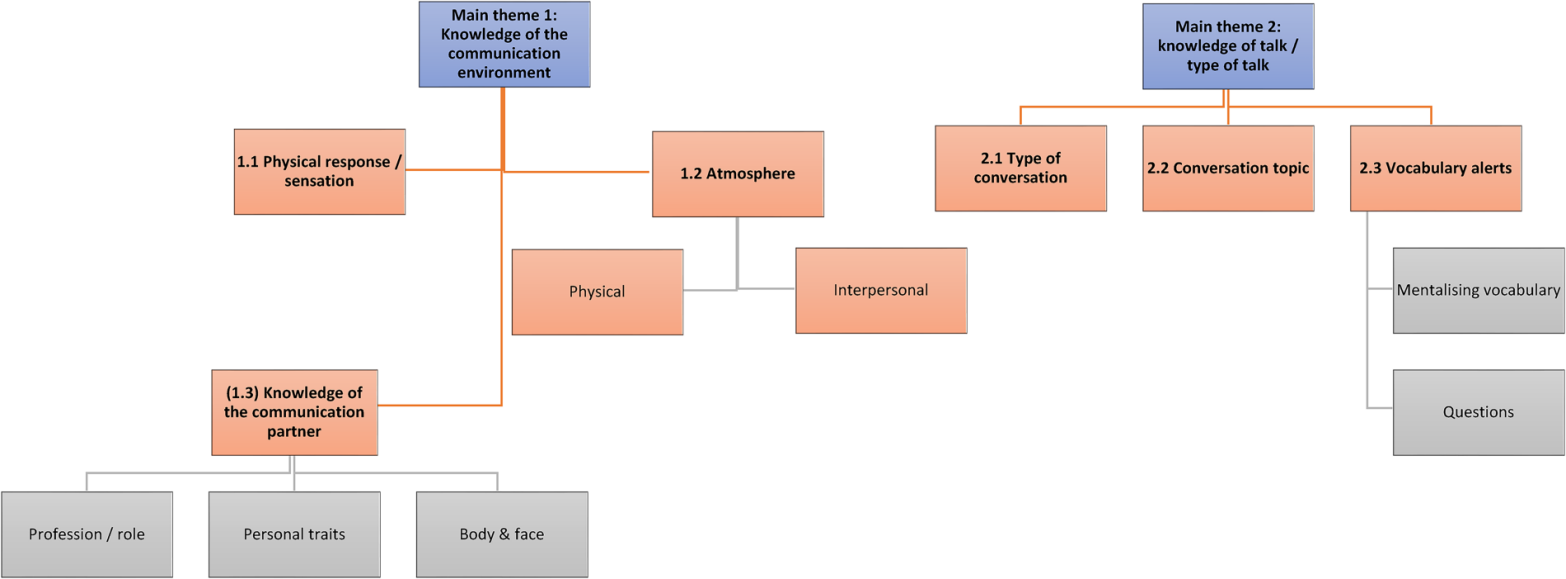
Thematic analysis map: main themes 1 and 2 and sub-themes.

The study explored what autistic participants know about, and find useful or unhelpful, in conversations. The overarching finding was that the autistic participants had existing useful knowledge about what was important and helpful to them personally in conversations, which is described in two main themes, and sub-themes, summarised in Figure 2. The main themes are (1) Knowledge of the communication environment and (2) Knowledge of talk / type of talk; both are explained and illustrated further below according to sub-themes.

### Main theme 1. Knowledge of the communication environment [the situation and the people in it]

#### Physical response or sensation

Autistic participants talked about awareness of a physical response or sensation within their own body when a conversation or interaction was not going well and could use this awareness as a trigger to use self-prompt strategies to make the situation better for themselves, for example Chloe recognised that:

‘…it [the conversation] did not go well…that was when my jaw started to stress out, … which does happen in every stressful situation’

When Chloe notices her jaw, she knows that something is not right for her and she uses this as a prompt to begin problem-solving. She gave the following example:

‘I was in the car on the way to the gym with Wyn, I could feel it going. We planned the meeting then it was sort of reducing.… problem-solving was the outcome.’

Cait described a feeling of a ‘cold arm’ while Carl described his ‘stomach swinging’. Ruth described a ‘sensation like I am stuck in the mud’ when things are difficult; she later shortened this to ‘stuck mode’ and used this feeling as a self-prompt, saying, ‘there's a little voice in my head that says you don't have to be in this situation, you need to get yourself out of it’. Lee became aware of his leg moving up and down as an indicator that something was not right for him and described this as a ‘leg to mouth message’, whereby ‘the leg tells me I need to stop and think’. All autistic participants described the physical sensations without attaching any mentalising words to describe an emotion being experienced by themselves at the time. They also reported that recognising and using a physical response or sensation as a prompt to think what to do next helped them to feel more in control in a social situation.

#### ‘Atmosphere’

This sub-theme uses Cait's word to describe her perception of the environment in which the interaction was taking place. Autistic participants reported that some environments were better for talking than others and discussed disliked aspects of a physical environment. For example, an environment with interruptions, unpredictable behaviour by others or noise was disliked by Cait and Lee. When asked to reflect on the first semi-structured interview, Lee said ‘it wasn't going well with all those interruptions’ (a phone ringing in a bag and a person entering unexpectedly), yet he had not specifically commented on these during the interview. Autistic participants had different preferences, for example, the ticking clock in a room was disliked by Lee, but, when Ruth was asked about this, she reported that the ticking clock did not concern her. Ruth says that she dislikes people talking loudly and now asks her friend to ‘turn her voice down a bit’ when she finds it too loud.

It was clear that all participants had different preferences in relation to the physical environment and knew what these were, even though they had not always previously talked about these. Participants also had different preferences, meaning that assumptions about the preferences of autistic people as a homogenous group should be avoided. Autistic participants reported that being more aware of disliked and preferred environments enabled them to indicate their dislikes to others and, where possible, to make changes to the environment.

The interpersonal environment was also important. All autistic participants reported that some people were easier to talk to than others. For example, Chloe identified that a person who talks too fast is not helpful to her, and Cait and Lee both disliked environments where many people were talking. Autistic participants identified that feeling listened to, or ‘having my say’ (Ruth), was important. This crucially linked to ‘power balance’; that is, a feeling of being ‘equal’ in the conversation and being able to have a say. Lee talked about the importance of ‘getting a good response’ from conversation partners and became more aware of where he did not get a ‘good response’.

#### Knowledge of the communication partner

Autistic participants used knowledge of people’s professions, personalities and usual face and body postures (when the person was known to them) to support their understanding of, and response to others, in conversations. For example, talking about her manager in the first semi-structured interview, Ruth described how her manager sounded bossy and had a stern face. Following learning about what she knows about people, she later reflected that ‘the manager had to be like this because she was in charge’, so her perceived bossiness no longer concerned Ruth. In other words, Ruth recognised the traits associated with the role of manager, making the manager predictable rather than worrying to her. Talking about his teacher, Lee said, ‘I don't like being told what to do’, but then, using knowledge of people linked to their profession, he recognised that ‘telling is the teacher's job’, again using knowledge about the role of a teacher to inform his expectations of that conversation.

Autistic participants also demonstrated knowledge of others’ personality, which was useful to them. For example, showing knowledge of her father's personality Ruth said:

‘Sometimes he does not switch off from his work mode, he has his work mode at home as well, so he will say ‘right Ruth, you do that, you do that’…. It doesn't bother me; I know my Dad and he has done it for years.’

Cait talked about joking with one of the managers at work, ‘because he likes to joke’, but would not joke with the other manager; and further commented that she would not joke with her Dad ‘because Dad doesn't do joking’. Cait likes to joke, so having this knowledge about other people and their response to joking enabled her to avoid joking with people who do not like this and so avoid an unsuccessful interaction from her perspective.

Autistic participants also had knowledge of individual traits and familiar body language of people known to them. For example, Lee commented that he knows not to approach his teacher when she puts her hands on her hair because it means ‘she is stressed and won't listen’. He says that once he learned this, the number of ‘bad conversations’ with his teacher reduced. Similarly, showing knowledge of the body language of her communication partner, Ruth said:

‘She has her normal face, but I know when she is busy or when she is flustered, she grabs her hair like this’. (*Ruth demonstrated running her hands through her hair*)

Cait became more aware and confident in knowing what someone would be ‘like’ following the interviews and reflective conversations and using this knowledge to feel more confident in a conversation. She described interactions with colleagues at work that would previously have caused her difficulty. For example, she reported that she now knew ‘what Jean was like’, and so her abrupt tone did not bother her anymore. Cait's communication partner commented that since Cait began to recognise what she knows about communication partners, Cait has been less anxious about conversations in general. During the study, Lee learned that he knows a good deal about people familiar to him, including how they may act and what to expect from them. Prior to the study, he had not realised that he had this useful knowledge.

### Main theme 2: knowledge of talk/type of talk

#### Type of conversation

Autistic participants labelled different types of conversation, including, for example: ‘bollocking’ conversation (Lee); ‘conclusion-solving’ (Chloe); and ‘help and advice’ (Ruth), and reported that knowing the type of conversation enabled them to know what to expect and to be prepared. For example, Lee talked about knowing when ‘to switch the brain on’ and Ruth said she knew she had to think ‘super hard’ in a ‘problem solving’ conversation.

Referring explicitly to type of conversation also enabled Ruth and her conversation partner to structure their conversations between themselves making clear, for example, when the ‘chit chat’ was finished and it was time for the ‘working out’. Ruth reported that this clarity helped her to ‘tune in’ to the conversation. Similarly, Lee and his communication partner were able to make clear when ‘banter’ was finished and it was time for an ‘agreeing’ conversation. These are good examples of how knowing the type of conversation enabled expectations for the conversation to be shared and so led to the possibility of a conversation being more successful. For example, Lee had an expectation based on experiences in a different education setting, that conversations requested by tutors would be ‘bollocking conversations’, which impacted on Lee's engagement in the conversation. However, when the tutor established the type of conversation at the start of the conversation, for example ‘an agreeing conversation’, (i.e., a problem-solving conversation) Lee was able to know what to expect and to participate more fully.

#### Conversation topic

Autistic participants reported that they liked to be clear about the conversation topic i.e., what would be talked about as well as the ‘type’ of conversation (above). For example, prior to having a conversation important to her, Chloe learned that is helpful to list exactly what she wants to talk about (for example, ‘the gym, the cost and the trainer’). Chloe and the conversation partner then both knew exactly what they were going to talk about. Lee commented that listing the conversation topics helped him to feel more in control of the conversation and to know when the conversation may be finished. Cait suggested that agreeing the topics of the conversation helped her and her communication partner during the study to stick to the topics, and finish talking about them, rather than divert to other topics, which could leave topics unfinished from Cait's perspective.

#### Vocabulary alerts

Analysis showed that most autistic participants experienced some difficulty when invited to talk about feelings. Examples included: (1) disliking the mixing of mentalising vocabulary, such as ‘stressed and anxious’ being used together as a single phrase (Ruth), and ‘angry and upset’ being used together (Carl); and (2) becoming hesitant in the response. For example, in response to ‘how did you feel?’ and then Carl usually providing a single word ‘bad’, or Cait suggesting it was hard to say and so not responding when asked about her feelings.

Personalised differences in understanding and use of a mentalising vocabulary were common to all autistic participants. Cait rarely used a mentalising vocabulary and suggested that she did not like to talk about feelings, Lee used some vocabulary to describe his feelings, but the meaning attached to the word was not always owned by him. For example, he used the word ‘meltdown’ apparently appropriately when asked to talk about his mental state in a difficult situation but, when asked to describe this, he said that it was his GP's word, not his. However, the autistic participants were able to describe their mental states in ways unique to them, for example Ruth used the words ‘stuck in the mud’ to describe a situation where she appeared to feel uneasy or unsure.

Autistic participants also identified that they found it more difficult to respond in conversations when sentences spoken to them were long or grammatically complicated. They commented on a dislike of some questions, particularly multiple questions (i.e., several questions asked one after the other in a single conversational turn), and ‘why’ questions. For example, Cait, Ruth, Chloe and Lee all commented that they disliked ‘why?’ questions or too many questions. Talking about ‘why?’ questions, Chloe said: ‘it pressures you to get the answer’ and ‘if no one's asking me a question, I can explain it without someone asking me why’. Ruth commented, ‘I didn't really want her to ask all the ‘why’ questions.’ However, autistic participants were able to contribute their ideas to other questions and at times in response to ‘why’ questions, suggesting further exploration of use of questions would be useful. Autistic participants reported that it was helpful for them to become more aware of words that they disliked so that they could let the communication partner know this. For example, Chloe developed a strategy with her communication partner to say ‘You know I don't like “why?” questions’ when asked these.

## Discussion

There is very little research that seeks to understand the experiences and knowledge of conversations from a strengths-based perspective of autistic adults; that is, from a starting point of what autistic people know and find useful rather than what other (usually non-autistic) people say they should learn or focus on. The current study aimed to learn about what was useful to autistic participants to know and do during conversations, so that they felt most able to contribute in ways that felt comfortable to them. The findings showed that autistic participants felt more able to contribute to a conversation when they had a feeling of control and knowledge of the conversation. They found it helpful to know about the communication partner and the communication environment, including the type of conversation (for example ‘conclusion solving’ or ‘banter’) and the topic of conversation (for example, ‘a conversation we had last week’, or ‘sexuality’). Autistic participants’ knowledge about themselves and their own responses in the conversation was also helpful.

Knowing the type and topic of conversation appeared to make the conversation more predictable for the autistic person, enabling them to anticipate the conversation structure, thus reducing uncertainty when entering the conversation. It is recognised that intolerance of uncertainty is associated with autism and may contribute to anxiety, and that desire for predictability is a part of intolerance of uncertainty (Stark et al., 2021). Being more certain about what to expect in the conversation may have reduced the demand for cognitive flexibility to work out and problem-solve what the conversation may be about and what may be expected. This, in turn, may reduce anxiety in the conversation since cognitive inflexibility may be linked to anxiety and low mood (Ozsivadjian et al., 2021). The autistic participants also used some trait knowledge i.e., knowledge of personality traits (what people known to them ‘are like’) as well as traits relating to professions (e.g., ‘what teachers do’), and the use of this trait knowledge seemed to support their predictive abilities. All the autistic participants in the present study reported that accessing and using knowledge of their communication partner was useful, saying how it enabled them to guess what the communication partner may do or say, hence reducing their uncertainty about the communication partner.

This aligns with the findings of Ramachandran et al. (2009) who investigated the ability of autistic adults to infer traits from descriptions of behaviour and reported trait inference to be a relative cognitive strength. It is recognised that autistic people often prefer structure (Ponnet et al., 2008), and this structure may be used to teach conversation skills (e.g., Doggett et al., 2013), and that certain types of conversation that may lack structure can be particularly difficult for autistic people, for example small talk (Trembath et al., 2012) and negotiation (Hochhauser et al., 2015). Consequently, the complementary use of explicit structure in the form of a stated agenda for (at least some) conversations and informal structure based on trait knowledge could be fruitful lines of enquiry to explore further in research and relatively straightforward adjustments to try out in practice in the meantime.

It was notable too that autistic participants used some mentalising words without being clear of their typically expected meaning and all used their own vocabulary to reflect how they may be feeling. There have been several studies exploring the link between alexithymia, defined as difficulty in identifying and describing own feelings (Nemiah, 1977), and autism (see Lartseva et al., 2015 for a review). It is suggested that approximately half of the autistic population experience alexithymia but the precise relationship between alexithymia and autism is little understood (Poquérusse et al., 2018). The findings of this study show that the autistic participants had some knowledge of their feelings but often used a unique vocabulary to describe these, suggesting that it is important to avoid normative expectations of understanding and use of mentalising words.

Further, all autistic participants described some physical response or physical sensation when they felt a conversation was not going well. The autistic participants had not previously been consciously aware of these experienced physical responses so had not realised the value of these as a self-prompt trigger to begin to use their cognitive problem-solving abilities, and thus to aim to take control of the environment or their situation so that they could feel better. Physiological arousal associated with anxiety is discussed by Bellini (2004) and by Trembath et al. (2012); it could be useful to explore autistic experience of physical response further and specifically, how the awareness of physical responses may be used by autistic people to recognise and manage an emotional response. This may be useful in removing any dependence on a vocabulary of emotions that may not make sense to themselves or, indeed, to others, to help the development of self-management strategies.

Autistic participants used idiosyncratic words or phrases, such as ‘conclusion solving’ to describe different types of conversations. These words and phrases were helpful because they were owned by the person and made sense to them and removed the need to learn someone else's vocabulary to describe conversation. It was also very helpful to the autistic participants when their words were accepted, understood, and then used with shared understanding by their communication partner. When the communication partner followed the lead of the autistic person, the autistic person reported a reduced risk of them being misunderstood and an increased likelihood that the conversation would feel more successful. We suggest this demonstrated respect for the autistic person's perspective and willingness by the communication partner to start from where the autistic person is, rather than imposing the normative non-autistic rules of communication so familiar in typical social skills training approaches (Bottema-Beutel et al., 2018). Accordingly, we propose there was also a shift in power from the usually more powerful and dominant stakeholder (the non-autistic majority) to the usually less powerful, more marginalised stakeholder (the autistic minority; Bottema-Beutel et al., 2018; Milton, 2012), which contributed to the feeling for the autistic person of being more in control of the conversation and, therefore, successful (on their own terms).

There are clear applications of these findings for Milton’s (2014, p. 794) discussion of ‘interactional expertise’ which, in simple terms, is ‘an understanding of the intentions and motives behind the actions of others’ (p. 798), such that those from one social group can interact successfully with others from a different social group. In this sense, Milton (2012, 2014) discusses autistic and non-autistic people as coming from two different social groups or ‘practice communities’ (p. 795), with concomitantly different social norms, expectations, and language practices. He argues that the gaining of interactional expertise by non-autistic people when communicating with autistic people is crucial for developing improved understanding, practice, and research that more authentically addresses the needs and perspectives of autistic people. This stance aligns also with the philosopher Bakhtin's theorising on the dialogic nature of existence and language in the sense that the ‘rules of encounter… are manifest only through dialogue with ‘other’ (White, 2013, p. 5). In other words, the:

‘meaning [of all interactions] is relative in the sense that it comes about only as a result of the relation between two bodies occupying *simultaneous but different* space’ (Holquist, 1990, p. 20, emphasis in original)

Given that the language practices of autistic people have been considered to be disordered or deficient for so long and, therefore, marginalised within practice and research (Bottema-Beutel et al., 2018; Milton, 2014) it does require a shift in power such that the ‘voices of the marginalized [sic] or silenced are promoted and respected’ (Kim, 2006, p. 6). Thus, our emphasis in this research on starting with the perspectives of autistic people to inform understandings about successful conversations is very much in line with this stance. In short, the findings show the Bakhtinian ‘dialogic principles’ (White, 2013, p. 5) that mattered to these autistic participants. Within this conceptual framing, there is also methodological strength and validity in observing and reflecting on conversations *with* autistic people in more naturalistic contexts to understand their discursive reality or ‘lived world’ (Davies, 2015, p. 45). Consequently, we argue that the study has ‘epistemic integrity’ (Bottema-Beutel et al., 2018, p. 961). Accordingly, the learning that can be taken from the present study is likely to be most applicable to those who work with autistic people in a range of settings, including researchers; professionals and practitioners from health, education and social care; and families. These findings could help them to be more effective and enabling communicators. Developing awareness and skills to be able to know what is helpful and unhelpful in a communication partner on an individual level could be an important component of guidance or training relating to development of effective strategies for communication that moves in valuable ways beyond the traditional confines of manualised social skills training approaches (Bottema-Beutel et al., 2018).

Starting with where autistic participants are is also vital for helping to make explicit what they may already know and for using that important knowledge for building a more shared understanding of the rules of encounter (Silver & Parsons, 2015), thereby starting to address the double empathy problem (Milton, 2012). Autistic participants reported that they were previously unaware that they had useful knowledge that could be used to inform their expectations and feeling of control of conversations. For example, Ruth commented: ‘Doing this [taking part in the research] has helped me a lot. I know more about what I know myself’. Further, their familiar communication partners within the dyad did not know that the autistic participants had such knowledge of people and situations, possibly reflecting negative assumptions of the capabilities of autistic people (Fletcher-Watson et al., 2019) or lack of awareness of metacognition (knowing about knowing) as an area worth exploring to identify supportive strategies (Sawyer et al., 2014).

## Limitations

The authors are not autistic and do not have the lived experiences of an autistic person and therefore cannot and do not speak for autistic people. However, gaining strong interactional expertise has been a lifelong endeavour across a range of professional roles and personal experiences and so, throughout the reporting of the findings, the intention is not to speak for the autistic participants but rather reflect what has been learned with and from them. Reflective practice and reflexivity were used alongside listening, to aim to hear the meaningful voices of the autistic participants and avoid any assumptions regarding their experiences.

This was a small-scale case study taking place over a relatively short period of time (4–12 months) and so there are inevitable limitations to the generalisation potential for learning. Although participants have themselves generalised their learning to other situations and to other communication partners since the end of the study, it is important to be aware of the heterogeneity of autism and the risks of assumptions and generalisation to other autistic people who may have differing abilities and preferences in relation to conversation. Heterogeneity also includes whether participants have co-occurring anxiety or depression; this information was not known about the current participants and this could help interpretation and may influence generalizability. Future research could explore more fully the experiences of those who report mental health concerns to better understand how communication preferences may be influenced. Further, the presentation of autism can change with time (Hobson, 2014) and much of the literature used to inform the study was based on young people, as there is relatively little relating to adults. Nevertheless, this study contributes to the literature by focusing on the communication of adults rather than children.

The timeframe of the study meant that the length of involvement with each dyad was limited. Anecdotally, three of the participants continued to use their learning after the close of the study, but a follow-up study to explore how this learning has been further sustained and developed (or not) would be useful. Lack of follow-up data in studies has been identified as a weakness in the study of social abilities (e.g., Gates et al., 2017) and so this is something that could usefully strengthen future research in this area.

Much of the reported learning during the study was based on self-report and questions have been raised about the validity of such measures, at least for very structured, targeted lines of questioning (e.g., Mazefsky et al., 2011). However, it was essential to the epistemological and ontological position of this research to hear the self-reports of autistic people, so care was exercised when drawing interpretations and conclusions from qualitative interviews (Sigstad & Garrels, 2018), and interpretations and conclusions were checked regularly with participants to ensure authenticity and trustworthiness. Further research could extend this line of enquiry to those who use alternative forms of communication (such as speech-generating devices) to explore whether and how communication mode makes a difference to experiences, preferences and understanding.

Autistic participants were invited to choose their communication partner to be involved in the study and each chose a person with whom they had regular important or significant conversations (from their perspective). The non-autistic participants were motivated to improve the quality and outcomes of their conversations with the autistic participants and so different findings could well arise with less motivated participants. Furthermore, exploration of how learning from this study could be applied in autistic peer-to-peer conversations would be useful.

## Conclusions

The findings from this study, grounded in the strengths and individual perspectives of autistic people as a starting point, provide insights into the ways that these autistic adults thought about conversations and what other people do, or do not do, that can help or hinder successful communication. The study also helped participants to identify and use previously unrecognised metacognitive abilities (what they already knew about conversations) within naturalistic interactive contexts. Use of these abilities gave a greater sense of empowerment for the autistic person within the conversation based on their accounts of their experiences. The findings from this study have valuable implications for staff training, for working with families and for learning by autistic adults.

## References

[bibr2-23969415221101113] Baghdadli A. Brisot J. Henry V. Michelon C. Soussana M. Rattaz C. Picot M. C. (2013). Social skills improvement in children with high-functioning autism: A pilot randomized controlled trial. European Child and Adolescent Psychiatry, 22(7), 433–442. 10.1007/s00787-013-0388-8 23417625

[bibr3-23969415221101113] Beardon L. Edmonds G. (2007). ASPECT consultancy report: A national report on the needs of adults with Asperger Syndrome. https://www.sheffield.ac.uk/polopoly_fs/,1…/file/ASPECT_Consultancy_report.pdf.

[bibr4-23969415221101113] Bejerot S. Eriksson J. M. Mörtberg E. (2014). Social anxiety in adult autism spectrum disorder. Psychiatry Research, 220(1–2), 705–707. 10.1016/j.psychres.2014.08.030 25200187

[bibr5-23969415221101113] Bellini S. (2004). Social skill deficits and anxiety in high-functioning adolescents with autism spectrum disorders. Focus on Autism and Other Developmental Disabilities, 19(2), 78–86. 10.1177/10883576040190020201

[bibr6-23969415221101113] Bishop-Fitzpatrick L. Minshew N. J. Eack S. M. (2014). A systematic review of psychosocial interventions for adults with autism spectrum disorders. In: Adolescents and adults with autism Spectrum disorders (pp. 315–327). Springer. 10.1007/s10803-012-1615-8 PMC3508309 22825929

[bibr7-23969415221101113] Bottema-Beutel K. Kapp S. K. Lester J. N. Sasson N. J. Hand B. N. (2021). Avoiding ableist language: suggestions for autism researchers. Autism in Adulthood, 3(1), 18–29. 10.1089/aut.2020.0014 PMC8992888 36601265

[bibr8-23969415221101113] Bottema-Beutel K. Park H. Kim S. Y. (2018). Commentary on social skills training curricula for individuals with ASD: social interaction, authenticity, and stigma. Journal of Autism and Developmental Disorders, 48(3), 953–964. 10.1007/s10803-017-3400-1 29170937

[bibr9-23969415221101113] Braun V. Clarke V. (2006). Using thematic analysis in psychology. Qualitative Research in Psychology, 3(2), 77–101. 10.1191/1478088706qp063oa

[bibr10-23969415221101113] Braun V. Clarke V. (2020). One size fits all? What counts as quality practice in (reflexive) thematic analysis? Qualitative Research in Psychology, 18(3), 328–352. 10.1080/14780887.2020.1769238

[bibr11-23969415221101113] Carrington S. Graham L. (2001). Perceptions of school by two teenage boys with Asperger syndrome and their mothers: A qualitative study. Autism, 5(1), 37–48. 10.1177/1362361301005001004 11708388

[bibr12-23969415221101113] Chevallier C. Huguet P. Happé F. George N. Conty L. (2013). Salient social cues are prioritized in autism spectrum disorders despite overall decrease in social attention. Journal of Autism and Developmental Disorders, 43(7), 1642–1651. 10.1007/s10803-012-1710-x 23138729

[bibr13-23969415221101113] Crane L. Adams F. Harper G. Welch J. Pellicano E. (2019). ‘Something needs to change’: Mental health experiences of young autistic adults in England. Autism, 23(2), 477–493. 10.1177/1362361318757048 29415558

[bibr14-23969415221101113] Cribb S. Kenny L. Pellicano E. (2019). ‘I definitely feel more in control of my life’: the perspectives of young autistic people and their parents on emerging adulthood. Autism, 23(7), 1765–1781. 10.1177/1362361319830029 30818981

[bibr15-23969415221101113] Davies K. (2015). The life and times of ‘Asperger’s Syndrome’: A Bakhtinian analysis of discourses and identities in sociocultural context. Doctoral thesis, The University of Queensland, Australia. https://espace.library.uq.edu.au/view/UQ:381703/s3106306_phd_submission.pdf?dsi_version=fe963825b96c8a34a54a8b9eaeaeed48

[bibr16-23969415221101113] Doggett R. A. Krasno A. M. Koegel L. K. Koegel R. L. (2013). Acquisition of multiple questions in the context of social conversation in children with autism. Journal of Autism and Developmental Disorders, 43(9), 2015–2025. 10.1007/s10803-012-1749-8 23292139 PMC3631576

[bibr17-23969415221101113] Fletcher-Watson S. Adams J. Brook K. Charman T. Crane L. Cusack J. Leekam S. Milton D. Parr J.R. Pellicano E., (2019). Making the future together: Shaping autism research through meaningful participation. Autism, 23(4), 943–953. 30095277 10.1177/1362361318786721 PMC6512245

[bibr18-23969415221101113] Flynn L. Healy O. (2012). A review of treatments for deficits in social skills and self-help skills in autism spectrum disorder. Research in Autism Spectrum Disorders, 6(1), 431–441. 10.1016/j.rasd.2011.06.016

[bibr19-23969415221101113] Frith U. (2012). The 38th Sir Frederick Bartlett lecture. Why we need cognitive explanations of autism. Quarterly Journal of Experimental Psychology, 65(11), 2073–2092. 10.1080/17470218.2012.697178 22906000

[bibr20-23969415221101113] Galanopoulos A. Robertson D. Spain D. Murphy C. (2014). Mental health and autism. National Autistic Society: Your autism magazine, 8.

[bibr21-23969415221101113] Gates J. A. Kang E. Lerner M. D. (2017). Efficacy of group social skills interventions for youth with autism spectrum disorder: A systematic review and meta-analysis. Clinical Psychology Review, 52, 164–181. 10.1016/j.cpr.2017.01.006 28130983 PMC5358101

[bibr22-23969415221101113] Grainger C. Williams D. M. Lind S. E. (2014). Metacognition, metamemory, and mindreading in high-functioning adults with autism spectrum disorder. Journal of Abnormal Psychology, 123(3), 650. 10.1037/a0036531 24955572

[bibr23-23969415221101113] Grandin T. (1995). Thinking in pictures: and other reports from my life with autism. Doubleday.

[bibr24-23969415221101113] Hobson R. P. (2014). The coherence of autism. Autism, 18(1), 6–16. 10.1177/1362361313497538 24151128

[bibr25-23969415221101113] Hochhauser M. Weiss P. L. Gal E. (2015). Negotiation strategies of adolescents with high-functioning autism spectrum disorder during social conflicts. Research in Autism Spectrum Disorders, 10, 7–14. 10.1016/j.rasd.2014.10.022

[bibr26-23969415221101113] Holquist M. (1990). Dialogism: bakhtin and his world. Routledge.

[bibr27-23969415221101113] Hull L. Petrides K. V. Allison C. Smith P. Baron-Cohen S. Lai M. C. Mandy W. (2017). ‘Putting on my best normal’: social camouflaging in adults with autism spectrum conditions. Journal of Autism and Developmental Disorders, 47(8), 2519–2534. 10.1007/s10803-017-3166-5 28527095 PMC5509825

[bibr28-23969415221101113] Humphrey N. Lewis S. (2008). Make me normal: the views and experiences of pupils on the autistic spectrum in mainstream secondary schools. Autism, 12(1), 23–46. 10.1177/1362361307085267 18178595

[bibr29-23969415221101113] Jackson L. (2003). Freaks, geeks and asperger syndrome. Jessica Kingsley.

[bibr30-23969415221101113] Jones R. S. Zahl A. Huws J. C. (2001). First-hand accounts of emotional experiences in autism: A qualitative analysis. Disability and Society, 16(3), 393–401. 10.1080/09687590120045950

[bibr31-23969415221101113] Kenny L. Hattersley C. Molins B. Buckley C. Povey C. Pellicano E. (2016). Which terms should be used to describe autism? Perspectives from the UK autism community. Autism, 20(4), 442–462. 10.1177/1362361315588200 26134030

[bibr32-23969415221101113] Kim J. H. (2006). For whom the school bell tolls: conflicting voices inside an alternative high school. International Journal of Education and the Arts, 7(6), 1–21. Retrieved from: 10.1177/1362361315588200.

[bibr33-23969415221101113] Knott F. Dunlop A. W. Mackay T. (2006). Living with ASD. How do children and their parents assess their difficulties with social interaction and social understanding? Autism**,** 10(6), 609–617. 10.1177/1362361306068510 17088276

[bibr34-23969415221101113] Lartseva A. Dijkstra T. Buitelaar J. K. (2015). Emotional language processing in autism spectrum disorders: A systematic review. Frontiers in Human Neuroscience, 8, 991. 10.3389/fnhum.2014.00991 25610383 PMC4285104

[bibr35-23969415221101113] Mazefsky C. A. Kao J. Oswald D. P. (2011). Preliminary evidence suggesting caution in the use of psychiatric self-report measures with adolescents with high-functioning autism spectrum disorders. Research in Autism Spectrum Disorders, 5(1), 164–174. 10.1016/j.rasd.2010.03.006 24013401 PMC3762501

[bibr36-23969415221101113] Miles M. B. Huberman A. M. Saldaña J. (2014). Qualitative data analysis: A methods sourcebook (3rd ed.). Sage.

[bibr37-23969415221101113] Milton D. E. (2012). On the ontological status of autism: The ‘double empathy problem’. Disability and Society, 27(6), 883–887. 10.1080/09687599.2012.710008

[bibr38-23969415221101113] Milton D. E. (2014). Autistic expertise: A critical reflection on the production of knowledge in autism studies. Autism, 18(7), 794–802. 10.1177/1362361314525281 24637428

[bibr39-23969415221101113] Milton D. E. Moon L. (2012). The normalisation agenda and the psycho-emotional disablement of autistic people. Autonomy, the Critical Journal of Interdisciplinary Autism Studies, 1(1), 1–12. Available at: http://www.larry-arnold.net/Autonomy/index.php/autonomy/article/view/AR3/21.

[bibr40-23969415221101113] Nemiah J. C. (1977). Alexithymia: theoretical considerations. Psychotherapy and Psychosomatics, 28(1–4), 199–206. 10.1159/000287064 609679

[bibr41-23969415221101113] Ozsivadjian A. Hollocks M. J. Magiati I. Happé F. Baird G. Absoud M. (2021). Is cognitive inflexibility a missing link? The role of cognitive inflexibility, alexithymia and intolerance of uncertainty in externalising and internalising behaviours in young people with autism spectrum disorder. Journal of Child Psychology and Psychiatry, 62(6), 715–724. 10.1111/jcpp.13295 32827150

[bibr42-23969415221101113] Pellicano E. Dinsmore A. Charman T. (2014). What should autism research focus upon? Community views and priorities from the United Kingdom. Autism, 18(7), 756–770. 10.1177/1362361314529627 24789871 PMC4230972

[bibr43-23969415221101113] Ponnet K. Buysse A. Roeyers H. De Clercq A. (2008). Mind-reading in young adults with ASD: does structure matter? Journal of Autism and Developmental Disorders, 38(5), 905–918. 10.1007/s10803-007-0462-5 17929156

[bibr44-23969415221101113] Poquérusse J. Pastore L. Dellantonio S. Esposito G. (2018). Alexithymia and autism spectrum disorder: A complex relationship. Frontiers in Psychology, 9, 1196. 10.3389/fpsyg.2018.01196 30065681 PMC6056680

[bibr45-23969415221101113] Ramachandran R. Mitchell P. Ropar D. (2009). Do individuals with autism spectrum disorders infer traits from behavior? Journal of Child Psychology and Psychiatry, 50(7), 871–878. 10.1111/j.1469-7610.2009.02053.x 19298473

[bibr46-23969415221101113] Rao P. A. Beidel D. C. Murray M. J. (2008). Social skills interventions for children with Asperger’s syndrome and high-functioning autism: A review and recommendations. Journal of Autism and Developmental Disorders, 38(2), 353–361. 10.1007/s10803-007-0402-4 17641962

[bibr47-23969415221101113] Reichow B. Steiner A. M. Volkmar F. (2013). Cochrane review: social skills groups for people aged 6 to 21 with autism spectrum disorders (ASD). Evidence-Based Child Health: A Cochrane Review Journal, 8(2), 266–315. 10.1002/ebch.1903 23877884

[bibr48-23969415221101113] Reichow B. Volkmar F. R. (2010). Social skills interventions for individuals with autism: evaluation for evidence-based practices within a best evidence synthesis framework. Journal of Autism and Developmental Disorders, 40(2), 149–166. 10.1007/s10803-009-0842-0 19655240

[bibr49-23969415221101113] Saldaña J. (2013). The coding manual for qualitative researchers (2nd ed.). Sage Publications.

[bibr50-23969415221101113] Sawyer A. C. Williamson P. Young R. (2014). Metacognitive processes in emotion recognition: are they different in adults with Asperger’s disorder? Journal of Autism and Developmental Disorders, 44(6), 1373–1382. 10.1007/s10803-013-1999-0 24272525

[bibr51-23969415221101113] Sigstad H. M. H. Garrels V. (2018). Facilitating qualitative research interviews for respondents with intellectual disability. European Journal of Special Needs Education, 33(5), 692–706. 10.1080/08856257.2017.1413802.

[bibr62-23969415221101113] Silver K. (2019). How can autistic adults be enabled to contribute their own thoughts and knowledge to significant conversations? [Doctoral Thesis, University of Southampton], p. 348. http://eprints.soton.ac.uk/id/eprint/433187

[bibr152-23969415221101113] Silver K. (2019). *How can autistic adults be enabled to contribute their own thoughts and knowledge to significant conversations?* [Doctoral Thesis, University of Southampton], p. 348. http://eprints.soton.ac.uk/id/eprint/433187.

[bibr52-23969415221101113] Silver K. M. Parsons S. (2015). Noticing the unusual: A self-prompt strategy for adults with autism. Advances in Autism, 1(2), 87–97. 10.1108/AIA-05-2015-0006

[bibr53-23969415221101113] Simonoff E. Pickles A. Charman T. Chandler S. Loucas T. Baird G. (2008). Psychiatric disorders in children with autism spectrum disorders: prevalence, comorbidity, and associated factors in a population-derived sample. Journal of the American Academy of Child and Adolescent Psychiatry, 47(8), 921–929. 10.1097/CHI.0b013e318179964f 18645422

[bibr54-23969415221101113] Spain D. Happé F. Johnston P. Campbell M. Sin J. Daly E. Ecker C. Anson M. Chaplin E. Glaser K. Mendez A., (2016). Social anxiety in adult males with autism spectrum disorders. Research in Autism Spectrum Disorders, 32, 13–23. 10.1016/j.rasd.2016.08.002

[bibr55-23969415221101113] Stark E. Stacey J. Mandy W. Kringelbach M. L. Happé F. (2021). Autistic cognition: charting routes to anxiety. Trends in Cognitive Sciences, 25(7), 571–581. 10.1016/j.tics.2021.03.014 33958281

[bibr56-23969415221101113] Trembath D. Germano C. Johanson G. Dissanayake C. (2012). The experience of anxiety in young adults with autism spectrum disorders. Focus on Autism and Other Developmental Disabilities, 27(4), 213–224. 10.1177/1088357612454916

[bibr57-23969415221101113] White J. (2013). Circles, borders and chronotopes: education at the boundary? Knowledge Cultures, 1(02), 145–169. Retrieved from: https://pesa.org.au/images/papers/2012-papers-English/E74.pdf.

[bibr58-23969415221101113] White S. Hill E. Winston J. Frith U. (2006). An islet of social ability in Asperger syndrome: judging social attributes from faces. Brain and Cognition, 61(1), 69–77. 10.1016/j.bandc.2005.12.007 16458402

[bibr59-23969415221101113] Williams D. M. (1992). Nobody nowhere. Transworld.

[bibr60-23969415221101113] Williams-White S. Keonig K. Scahill L. (2007). Social skills development in children with autism spectrum disorders: A review of the intervention research. Journal of Autism and Developmental Disorders, 37(10), 1858–1868. 10.1007/s10803-006-0320-x 17195104

[bibr61-23969415221101113] Wittemeyer K. English A. Jones G. Lyn-Cook L. Milton D. (2015). Schools autism competency framework. Autism Education Trust Competency Framework. https://www.spectrumasd.org/wp-content/uploads/2019/10/AET_CompetencyFramework_22012016-3.pdf

